# Th1 and Th17 but no Th2-related cytokine spectrum in the cerebrospinal fluid of children with *Borrelia*-related facial nerve palsy

**DOI:** 10.1186/2045-8118-10-30

**Published:** 2013-10-04

**Authors:** Zuzana Liba, Jana Kayserova, Vladimir Komarek

**Affiliations:** 1 Department of Pediatric Neurology, University Hospital Motol, Prague, Czech Republic; 2 Department of Immunology, University Hospital Motol, Prague, Czech Republic

**Keywords:** Children, Facial palsy, Neuroborreliosis, Cerebrospinal fluid, Cytokine, IL-4, IL-15, IL-17

## Abstract

**Background:**

Chemokines and cytokines in cerebrospinal fluid (CSF) and serum have been extensively studied in adults with neuroborreliosis (NB), whereas there are limited data about the pediatric population. In adults, T helper type 1 (Th1) and Th17-related cytokines were observed during acute NB. In children, the Th2 response is thought to moderate the disease course. The aim of this study was to determine the chemokine-cytokine profile in children with acute NB displaying *Borrelia*-related peripheral facial nerve palsy (PFNP).

**Methods:**

Luminex multiple bead technology was used for the detection of twelve cytokines and chemokines in the CSF and serum of three groups: 1) children with *Borrelia*-related PFNP (B_PFNP_); 2) children with non-borrelial “idiopathic” PFNP (NI_PFNP_); and 3) age-related controls.

**Results:**

In B_PFNP_, cytokines-chemokines related to a non-specific pro-inflammatory activity and specific Th1/Th17 responses were detected in CSF, and elevated IL-7 and IL-10 levels were observed in serum and CSF compared to NI_PFNP_ and to controls. In NI_PFNP_, CSF findings were similar to controls; however, higher levels of IL-7 and MCP-1 were observed in serum. Higher IL-8, IL-15 and MCP-1 levels were detected in CSF compared to serum in all groups. MCP-1 and IL-8 levels in CSF were strikingly higher in B_PFNP_ compared to the other two groups, while IL-15 levels in CSF showed no difference. In addition, in controls, increased IL-4 level was found in CSF compared to serum.

**Conclusion:**

The chemokine-cytokine profile in the CSF of children with acute NB was similar to previous studies in adults. Our data suggests that higher levels of IL-4, IL-15 and MCP-1 levels in CSF compared to serum in controls might represent a potentially protective cytokine milieu in the CNS compartment.

## Background

Neuroborreliosis (NB) is characterized by neurologic involvement during infection with the complex of *Borrelia burgdorferi* sensu lato (*Bb*) [1]. The clinical manifestation and clinical course of NB is different in children compared to adults [2]. Cranial neuropathy including the facial nerve is the most common manifestation of acute NB in the pediatric population [3,4]. The disease course seems to have a better prognosis and chronic NB is rarely observed in children [4].

*Borrelia*-species are facultative intracellular pathogens that are able to survive in the extracellular matrix [1]. All of the known types of immune response (T helper (Th) type 1, Th2 and Th17) as well as regulatory mechanisms are involved in *Bb*-induced inflammation [5-9]. Increased CD8^+^ interferon gamma (IFN-γ) –producing T cells were described in NB patients [7]. A strong Th1 inflammatory response, represented by production of IFN-γ and tumor necrosis factor alpha (TNF-α), is required for successful *Bb* eradication [7,8]. The Th2 immune response, represented by interleukin (IL)-4 and IL-13, may inhibit the potentially harmful effect of the Th1 response [5]. The benign course of NB in children was associated with elevated secretion of interleukin (IL)-4 in the CSF [8-10]. Recently, the Th17 response, represented by IL-17, has been observed in some cases of confirmed NB, and IL-17 has been suggested as an important mediator of *Bb*-induced immunopathology [11,12]. Increased levels of IL-17 were associated with more pronounced pleocytosis and fatigue in adults but not in children [12].

Cytokines have been extensively studied in adults with NB, whereas there are limited data about the pediatric population [6-11]. The aim of this study was to determine the concentrations of chemokines and cytokines (IFN-γ, IL-2, IL-4, IL-6, IL-7, IL-8, IL-10, IL-13, IL-15, IL-17, MCP-1 and TNF-α) in CSF and serum of children with *Borrelia*-related peripheral facial nerve palsy (PFNP) and to compare these data to children with non-borrelial “idiopathic” PFNP and to age-related controls.

## Methods

### Subjects

Thirty-four children with PFNP treated in the Pediatric Neurology Department, University Hospital Motol, Prague, Czech Republic, were included in the study: 20 children with *Borrelia*-related PFNP (B_PFNP_) and 14 children with non-inflammatory “idiopathic” PFNP (NI_PFNP_). All underwent lumbar puncture and serum withdrawal within five days of PFNP diagnosis (median time three days). All children with proven *Borrelia*-related facial palsy (see below) were treated with ceftriaxone intravenously (21 days, dose 50 mg/kg/day) [13]. A control group consisting of 23 age-matched children without PFNP, underwent lumbar puncture and serum withdrawal to exclude neuroinfection. Their symptoms were as follows: headache (n = 12), anomaly of dura mater (n = 2), abnormal gait (n = 1), vertebral algic syndrome (n = 1), orthostatic collapses (n = 1), paresthesia (n = 1), visual impairment (n = 1), sphincter problems (n = 1), sleep myoclonus (n = 1), macrocephaly (n = 1), cerebral palsy (n = 1). Objective neurological examination, magnetic resonance (MR) imaging and analysis of CSF and serum showed normal results. For group specifications, see Table 1. The project was approved by the local Ethical Committee and the University Hospital Motol. Informed consent was obtained from parents of all participants.

**Table 1:** Characteristics of the patient groups

	**B**_**PFNP **_**(n = 20)**	**NI**_**PFNP **_**(n = 14)**	**Controls (n = 23)**
**Age**	7.75	9.6	10
**(median, range)**	(4.2-14)	(2.2-17)	(0.5-17)
**Sex ratio**	11 : 9	7 : 7	12 : 11
**(F:M)**			
**Leukocytes in CSF**	75***	1	1
(median, range) [×10^-6^/L]	(6-444)	(0-4)	(0-5)
**Protein in CSF**	0.33****	0.16	0.18
(median, range) [g/L]	(0.14-0,97)	(0.13-0.34)	(0.12-0.32)
**Q**_**alb**_	6.0***	2.8	3.1
	(2.3-11.4)	(2.1-5.7)	(2-5.5)
***Borrelia*-specific-Ab in CSF**	+	-	-

B_PFNP_ children with *Borrelia*-related facial palsy; NI_PFNP_ children with non-inflammatory idiopathic facial palsy; Q_alb_ = CSF_alb_[g/L]/serum_alb_[g/L] × 10^3.^ P-values are indicated: *** p < 0,0005; **** p < 0,0001.

### Routine analysis and diagnostic process

CSF and serum were routinely examined in the Laboratory of CSF and in the Laboratory of Microbiology in University Hospital Motol. Cell count, protein level, blood-brain barrier (BBB) status, level of immunoglobulin (Ig) G and IgM were evaluated by standard methods. Pleocytosis was defined as total cell count in CSF > 5 × 10^6^/L. Status of the BBB was determined by albumin ratio Qalb (Qalb = albumin CSF [g/L] / albumin serum × 10^3^ [g/L]; Qalb > 5 determinates BBB deficiency [14]. The normal IgG level determined in the laboratory was < 0.040 g/L in CSF and 3.6 – 11.9 g/L in serum. The normal IgM level was < 0.0012 g/L in CSF and 0.6- 2.5 g/L in serum [14,15]. In cases of elevated levels of Ig in CSF, Reiber’s formula for intrathecal production of IgG and/or IgM was used [16].

The presence of antibodies against *Bb* was determined by using the enzyme-linked-immunosorbent-assay (ELISA) and western-blot (WB) for verification. Furthermore, CSF and serum were tested for the presence of specific antibodies against herpes-simplex virus, varicella-zoster virus, Epstein-Barr virus and the virus of tick-born encephalitis.

*Borrelia*-related peripheral facial nerve palsy (B_PFNP_) was diagnosed when there was pleocytosis in CSF and *Borrelia*-specific antibodies were present [12]. In case of PFNP with normal CSF and no specific antibody production, high-resolution computer tomography of pyramidal bones (HRCT) and MR imaging of the brain were performed. Idiopathic PFPN was diagnosed when all these were normal (NI_PFNP_).

After routine analysis all samples were stored at -20°C and thawed once before chemokine and cytokine analyses.

### Chemokine and cytokine detection

Chemokine and cytokine concentrations in CSF and serum were measured by Luminex multiple bead technology and software according to the manufacturer’s instructions (Customized kit, Milliplex Human Cytokine/Chemokine Panel, Millipore Corporation, Billerica, MA, USA). Chemokines IL-8, MCP-1 and cytokines IFN-γ, IL-2, IL-4, IL-6, IL-7, IL-10, IL-13, IL-15, IL-17 and TNF-α were determined in the three previously-characterized groups.

### Statistical analysis

Statistical analyses were performed using GraphPad PRISM, version 6.0. Non-parametric tests were used. Kruskal-Wallis ANOVA was performed to compare multiple study groups, and the Mann-Whitney test was applied as a post-hoc test. Wilcoxon signed ranked test was used for pair analysis of CSF and serum in each group. Correlations between parameters were calculated using Spearman correlation.

## Results

White cell count and protein level in CSF were significantly increased in the B_PFNP_ group compared to the other groups (Table 1). Both parameters correlated positively with albumin ratio (Q_alb,_ data not shown).

### CSF

**Figure 1:**
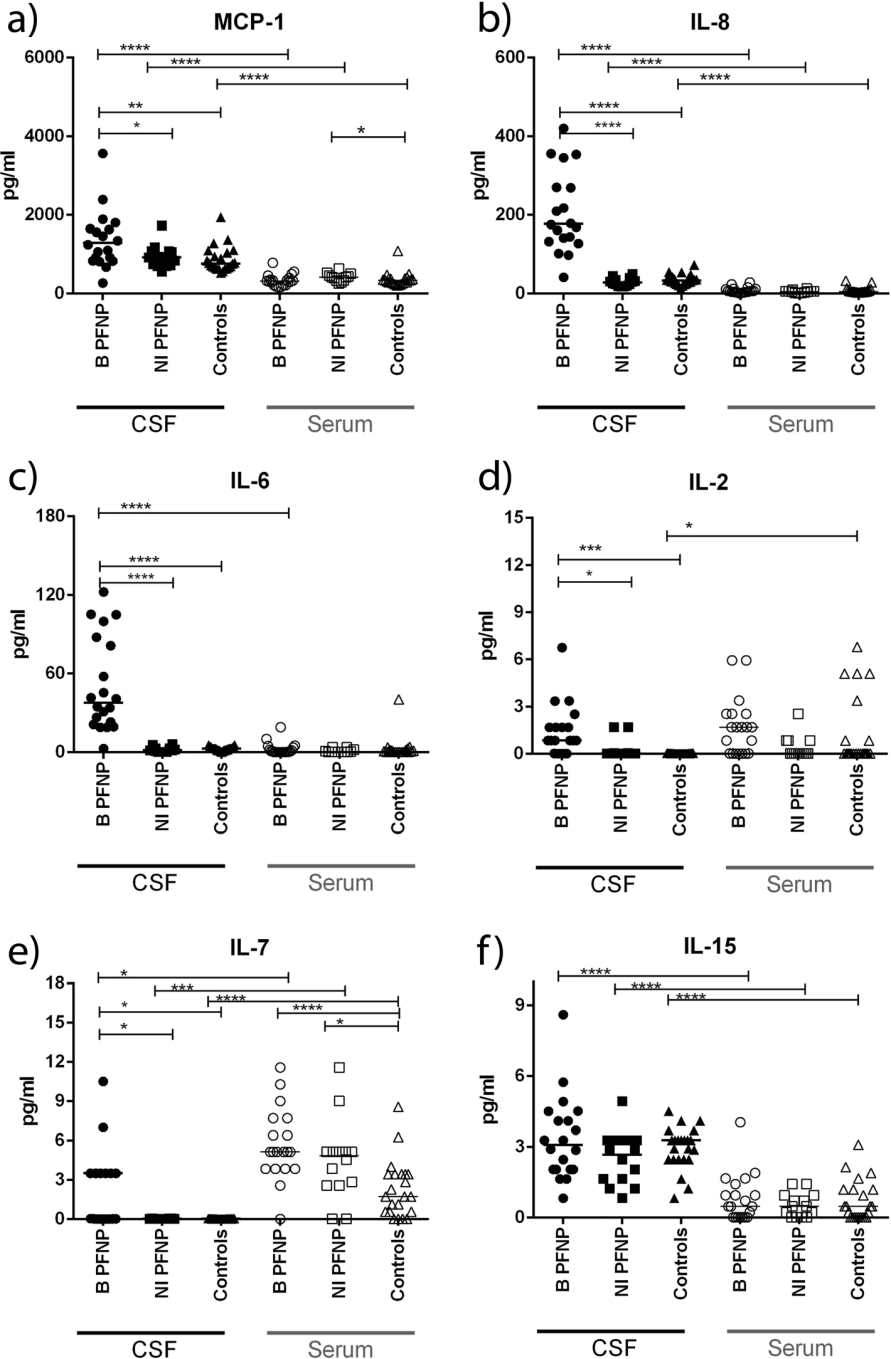
**Concentrations of chemokines and cytokines in cerebrospinal fluid (CSF) and serum: a-b)** MCP-1 and IL-8 chemokines and **c)** IL-6 non-specific pro-inflammatory cytokine were strikingly increased in CSF of B_PFNP_, **d**, **e)** IL-2 and IL-7 cytokines important for T and B cell proliferation and function were increased in CSF of B_PFNP_ (IL-2, IL-7) and in serum of B_PFNP_ and NI_PFNP_ (IL-7), **f)** IL-15 cytokine was increased in CSF compared to serum in all groups; B_PFNP_ (n = 20) children with *Borrelia*-related facial palsy; NI_PFNP_ (n = 14) children with non-inflammatory “idiopathic” facial palsy“; Controls (n = 23); P-values are indicated: * p < 0,05; **p < 0,005; *** p < 0,0005; **** p < 0,0001.

**Figure 2:**
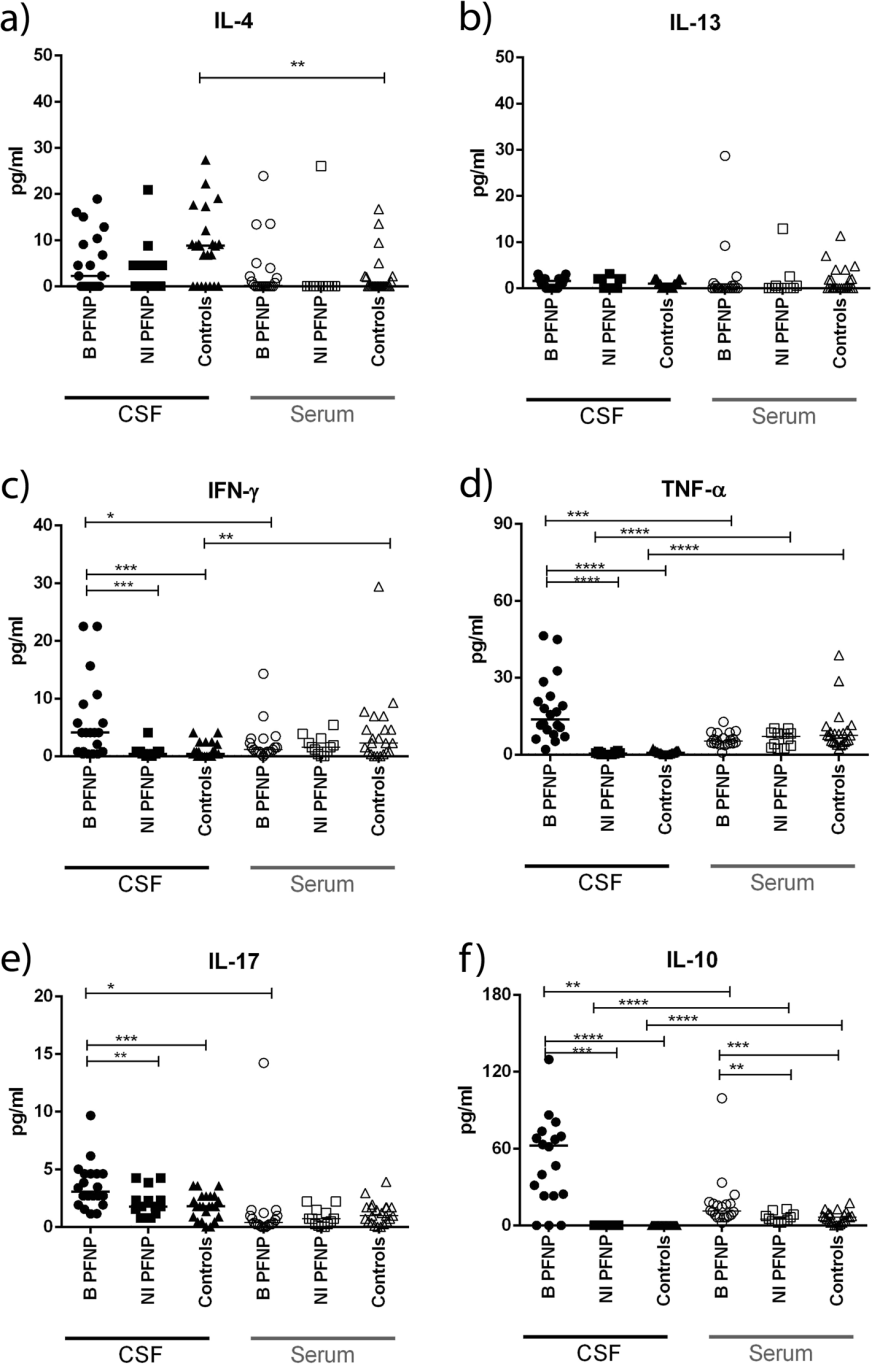
**Concentrations of cytokines related to the adaptive immunity in cerebrospinal fluid (CSF) and serum: a**-**b)** IL-4 and IL-13 /Th2 related cytokines – only IL-4 was increased in CSF of controls in comparison to serum, **c**-**f)** IFN-γ and TNF-α /Th1 related cytokines and IL-17/ Th17 related cytokine were strikingly increased in CSF of B_PFNP_, **f)** IL-10 regulatory cytokine was strikingly increased in CSF and serum of B_PFNP_; B_PFNP_ (n = 20) children with *Borrelia*-related facial palsy; NI_PFNP_ (n = 14) children with non-inflammatory “idiopathic” facial palsy “; Controls (n = 23); P-values are indicated: * p < 0,05; ** p < 0,005; *** p < 0,0005; **** p < 0,0001.

Significantly increased chemokines (MCP-1, IL-8) and cytokines (IL-2, IL-6, IL-7, IL-10, IL-17, IFN-γ and TNF-α) were detected in B_PFNP_ in comparison to CSF levels in the other groups (Figures 1a-f and 2c-f).

### Serum

Increased levels of IL-7 and IL-10 were detected in B_PFNP_ compared to serum levels in controls. Serum level of IL-10 in B_PFNP_ was also significantly higher than in the NI_PFNP_ group. In NI_PFNP_, increased levels of MCP-1 and IL-7 were detected in comparison to controls (Figures 1a, e and 2f).

### CSF vs. serum

In all groups, significantly higher levels of IL-8, IL-15 and MCP-1 were detected in CSF compared to serum. Additionally, significantly higher IL-4 level in CSF compared to serum was detected in controls (Figure 2a). IL-13 levels did not differ between any groups (Figure 2b).

All children with NB had complete clinical recovery from facial palsy within 21 days of the treatment onset.

## Discussion

The detection of chemokines and cytokines in CSF and serum during acute NB indicated that inflammation was localized mainly in the CNS compartment with an imprint in peripheral blood. Similar to the published data in adults with an early stage of acute NB, innate pro-inflammatory and specific Th1 and Th17-related cytokines were detected in CSF of children with *Borrelia*-related PFNP while IL-4/Th2-related cytokine was not [6-8,12].

As was previously described, antigen specific T cells are recruited to the CNS from peripheral blood once they are educated in the lymph nodes [17,18]. In accordance with the involvement of adaptive immunity during acute NB, we could observe increased IL-7 level in serum and IL-2 and IL-7 levels in CSF [19]. Increased level of IL-2 in CSF and not in serum in NB patients reflects a general T cell activation in the CNS [7].

Moreover, we detected raised levels of IL-10 in CSF and serum of children with acute NB. IL-10 is a cytokine with pleiotropic effects but mainly exerts a strong inhibitory function and prevents tissue damage [20]. *Bb* species are able to induce IL-10 production and thus regulate the immune response to their advantage [1,19]. Nevertheless, IL-10 is also able to promote cytotoxic T cell activity and antibody production [7]. So far, we could only speculate on whether increased IL-10 level might help in effective resolution of *Bb-*infection.

There is a growing body of evidence that less harmful immune responses are preserved in the CNS and that the intraparenchymal CNS environment is anti-inflammatory [17,18]. Certain cytokines, such as IL-4 and IL-15, have been recently studied in the context of neuroinflammation and seem to have a neuroprotective effect in animal models [21-23]. We detected a higher level of IL-15 in the CSF in all groups compared to serum. Additionally, we observed a higher CSF level of IL-4 in comparison to serum in the control group. In contrast, IL-4 in CSF of children with *Borrelia*-related PFNP was not increased compared to serum in our study. That could be caused by the fact that the samples were collected at a very early stage of NB (no more than five days after the neurologic symptoms had occurred). Our findings are in accordance with the previous study, in which early increased secretion of *Borrelia*-specific IFN-γ was observed and subsequent up-regulation of IL-4 in CSF was associated with non-chronic NB [8]. The potential regulatory role of IL-4 (Th2) in Th1-mediated pathologies is widely discussed. However, MCP-1 also seems to support a Th2 response [24]. Furthermore, the role of MCP-1 and IL-8 in brain function and development has been recently observed in animal models [25].

## Conclusion

Despite the slightly different clinical course of acute NB in children, the chemokine and cytokine production in CSF was similar to that of adults. Our data suggests that higher levels of IL-4, IL-15, and MCP-1 in CSF might represent a potentially protective cytokine milieu in the CNS compartment [20-25]. More studies have to be done to clarify the cytokine environment in the CNS and its role in health and disease.

## Competing interests

The authors declare that they have no competing interests.

## Authors’ contributions

ZL is the principal investigator for this project; she conceptualized and designed the project, collected CSF and serum samples, analyzed the results, drafted the manuscript and prepared it for publication. JK performed the laboratory work with sample handling and data acquisition from the Luminex technology and software; she supervised the analysis and intellectual content of the manuscript. VK supervised the clinical data and assisted in the draft of the manuscript. All authors read and approved the final manuscript.
